# Facile Synthesis of Nitrogen-Doped Carbon Dots from Lignocellulosic Waste

**DOI:** 10.3390/nano9101500

**Published:** 2019-10-22

**Authors:** Mohammed Abdullah Issa, Zurina Z. Abidin, Shafreeza Sobri, Suraya Rashid, Mohd Adzir Mahdi, Nor Azowa Ibrahim, Musa Y. Pudza

**Affiliations:** 1 Department of Chemical and Environmental Engineering, Faculty of Engineering, Universiti Putra Malaysia, 43400 UPM Serdang, Malaysia; mohbaghdadi1@yahoo.com (M.A.I.); shafreeza@upm.edu.my (S.S.); suraya_ar@upm.edu.my (S.R.); pudzamusa@gmail.com (M.Y.P.); 2 Department of Computer & Communications Systems Engineering, Faculty of Engineering, Universiti Putra Malaysia, 43400 UPM Serdang, Malaysia; mam@upm.edu.my; 3 Department of Chemistry, Faculty of Science, Universiti Putra Malaysia, 43400 Serdang, Malaysia; norazwa@upm.edu.my

**Keywords:** carbon dots, N-doped carbon dots, fluorescent probe, Cu^2+^ sensor, composite film

## Abstract

The current research mainly focuses on transforming low-quality waste into value-added nanomaterials and investigating various ways of utilising them. The hydrothermal preparation of highly fluorescent N-doped carbon dots (N–CDs) was obtained from the carboxymethylcellulose (CMC) of oil palm empty fruit bunches and linear-structured polyethyleneimines (LPEI). Transmission electron microscopy (TEM) analysis showed that the obtained N–CDs had an average size of 3.4 nm. The N–CDs were monodispersed in aqueous solution and were strongly fluorescent under the irradiation of ultra-violet light. A detailed description of the morphology and shape was established using Fourier transform infrared (FTIR) spectroscopy and X-ray photoelectron spectroscopy (XPS). It was shown that LPEI were successfully tuned the fluorescence (PL) properties of CDs in both the intrinsic and surface electronic structures, and enhanced the quantum yield (QY) up to 44%. The obtained N–CDs exhibited remarkable PL stability, long lifetime and pH-dependence behaviour, with the excitation/emission maxima of 350/465.5 nm. Impressively, PL enhancement and blue-shifted emission could be seen with the dilution of the original N–CDs solution. The obtained N–CDs were further applied as fluorescent probe for the identification of Cu^2+^ in aqueous media. The mechanism could be attributed to the particularly high thermodynamic affinity of Cu^2+^ for the N-chelate groups over the surface of N–CDs and the fast metal-to-ligand binding kinetics. The linear relationship between the relative quenching rate and the concentration of Cu^2+^ were applied between 1–30 µM, with a detection limit of 0.93 µM. The fluorescent probe was successfully applied for the detection of Cu^2+^ in real water. Moreover, a solid-state film of N–CDs was prepared in the presence of poly (vinyl alcohol) (PVA) polymer and found to be stable even after 72-h of continuous irradiation to UV-lamp. In contrast to the aqueous N–CDs, the composite film showed only an excitation independent property, with enhanced PL QY of around 47%. Due to the strong and stable emission nature of N–CDs in both aqueous and solid conditions, the obtained N–CDs are ideal for reducing the overall preparation costs and applying them for various biological and environmental applications in the future.

## 1. Introduction

Carbon dots (CDs), a new member of the fluorescent carbon nanostructures family, with sizes of less than 10 nm, have drawn considerable attention among researchers since their first fundamental report [1]. CDs consist of a graphitic or amorphous carbon core coated with oxygen-functionalities, polymers and other active groups with respect to synthesis condition and surface chemistry [2]. In contrast to organic dyes and traditional semiconductor quantum dots, CDs possess remarkable properties, including tuneable photoluminescence, chemical inertness, efficient production and good biocompatibility [3,4,5]. Due to these beneficial characteristics, they are useful in the areas of chemical sensing, bio-imaging, photocatalysis, and wastewater treatment [6,7].

Many methods, including the arc-discharge approach [8], electrochemical oxidation [9], microwave treatment [10] and hydrothermal route [11], with a large diversity of carbonaceous precursors, have been recently established for the production of CDs. As summarized in Table S1, the usage of carbon-based bulk materials, such as candle soot [12], activated carbon [13] and graphitic rods [14] resulted in low quantum yield (QY). Thus, a broad range of CDs have been synthesized using small molecules that serve as single precursors [15,16,17]. However, CDs synthesized from sole small molecules show weaker fluorescence (PL) compared to those from dual small precursors. Dual precursors, including the usage of cellulose/urea [18], glucose/polyethyleneimine (PEI) [19] and microcrystalline cellulose/ethylenediamine (EDA) [20] were used for the production of N–CDs, in which one serving as the carbon source, the other as nitrogen source or passivation agent. An N-doping source, also known as an electron donator, has been shown to be the most convenient element on the stimulating of electronic states of the carbonaceous material by introducing new energy states corresponding to the N-dopants [5]. In spite of their promising optical properties and low-toxic behaviour, several drawbacks limit their analytical applications, such as long duration synthesis processes from 7–72 h [16,19,20,21,22,23,24,25,26,27,28,29,30], low quantum yield of using sole precursors [10,14,19,23,24,27,29,30,31,32,33,34,35,36,37,38,39,40,41,42,43,44,45,46,47], or efficient quantum yield along with long duration synthesis process even after doping CDs with various heteroatoms [16,20,21,22,25,26]. Since the obtained QY is highly dependent on the particle size, choice of dopant, influence of the passivating species, pH, time, temperature, nature of the solvent, several shortcomings such as PL origin and the corresponding interaction variables in production are still controversial and need to be further discussed. Moreover, the ease of the processing and no need for high temperature and long duration synthesis processes is of great interest.

Water pollution by metal ions released by anthropogenic activities has become a worldwide issue owing to their severe threats to human health and environment [48,49]. One should note that Cu^2+^ is necessary in significant physiological functions. Nevertheless, excessive doses of Cu^2+^ in the human body can induce an acknowledged risk of diseases, including Menkes, Prion, Wilsons and Alzheimer’s, and so on [50]. Moreover, copper is highly exploited by industries, environment, and domestic functions. Therefore, it would be sensible to develop highly fluorescent N–CDs in a significantly short synthesis time as well as to improve the sensitive detection of copper ions in aqueous media.

In the present work, we demonstrated the use of low-cost wastes of carboxymethylcellulose (CMC) as the carbon material for the production of fluorescent CDs since various physical characteristics of CMC resulted in different degrees of polymerization. Linear-structured polyethyleneimine (LPEI) was selected as the N-doping source/surface passivation agent in an attempt to enhance the PL emission of CDs. LPEI polymer has been widely used for various fields such as solar cells, light emitting diodes, transistors owing to its polycationic character, which can provide CDs with amine groups with the ability to recognize analytes. The PL QY has been optimized by studying the effects of reaction temperature, time and LPEI dosage on the optical characteristics of N–CDs. The optimized N–CDs were thoroughly characterized using various analytical techniques, and their fluorescence stability in terms of LPEI–CDs concentration, pH effect, illumination time, ionic strength, surface charge and storage time was studied. The as-prepared N–CDs with bright greenish emission have shown a fluorescence QY of 44% which was found to be six times higher than the undoped CDs. The N–CDs exhibited both excitation dependence/independence emission and excellent stability for more than six months without any precipitation. Moreover, both PL emission enhancement and blue-shifted of emission maxima were observed with a decreasing concentration of N–CDs. The fluorescent probe was successfully applied for the determination of Cu^2+^ in aqueous solution, and a possible quenching mechanism was thoroughly studied. Fluorescent composite film was also prepared by the incorporating N–CDs into poly (vinyl alcohol) (PVA). Compared to the N–CDs solution, the solid film displayed higher QY (47%) with only an excitation-independent wavelength feature.

## 2. Materials and Methods

### 2.1. Reagents

CMCs were purchased from Waris Nove Company, Balok, Malaysia. The analytical-grade of LPEI (Mn ~5000) and quinine sulphate were obtained from Sigma-Aldrich (St. Louis, MO, USA). MgSO_4_, MnSO_4_, ZnSO_4_, CaCl_2_, CdCl_2_, HgCl_2_, FeCl_3_, FeCl_2_, PbNO_3_, NaOH, HCl, NaCl, AlCl_3_, KCl, BaCl_2_, LiCl, AgNO_3_, CoCl_2_, NiCl_2_, CuNO_3_ and PVA were purchased from R and M marketing, Selangor, Malaysia. Throughout the preparation of the solution, deionised water (DI) with a resistivity of 18.25 MΩ cm was used.

### 2.2. Preparation of N–CDs

Fine carboxymethylcellulose, 0.1 g, and a suitable amount of LPEI were added into 25 mL of DI water. Ultrasonication was the method used in this stage so the LPEI was added uniformly. Once they were mixed together, they were added into a 50 mL Teflon-lined stainless-steel reactor. The mixture was then sealed and heated at 260 °C for 2 h in an oven. The solution was cooled down at room temperature and then centrifuged at 10,000 rpm for 12 min so that a free-dark carbonaceous material could be obtained. The final solution was purified through vacuum filtration (0.22 µm) to remove the precipitate. It was then placed into a dialysis membrane (1 kDa) so that the contents of salt ions were eliminated. Similar procedures, except the addition of LPEI, were conducted for the preparation of the undoped carbon dots as a blank solution sample.

### 2.3. Instrumentation

Transmission electron microscopy (TEM) and high-resolution TEM (HRTEM) readings were carried out using A Tecnai G2 F20 electron microscope, with an acceleration voltage of 200 kV. The Fourier transform infrared (FTIR) (Thermo Nicolet FTIR spectrometer of 4 cm^−1^ resolution) was measured with KBr as a standard within the range from 650 to 4000 cm^−1^. UV–vis spectra were recorded with a spectral range of 200–800 nm using Shimadzu UV-1800 Spectrophotometer (Shimadzu Corporation, Tokyo, Japan). PL studies were measured in quartz cuvettes with 1 cm path length using LS 55 Fluorescence Spectrometer (PerkinElmer, Waltham, MA, USA) with a slit width of 15 and 5 nm for excitation and emission, respectively and a scan rate of 240 nm/min. X-ray photoelectron spectra (XPS) (Physical Electronics PHI 5400 spectrometer, Mimos Semiconductors, Kuala Lumpur, Malaysia) were measured using Al-Ka radiation (hν=1486.6 eV). Prior to de-convolution, charge correction was established at C_1s_ by setting binding energies of C–C and C–H at 284.8 eV. The pH values were obtained by a PB-10 pH-meter (Beijing Sartorius Instruments Co. Ltd., Beijing, China) and zeta potential study of N–CDs was carried out using Zetasizer Nano ZS (Malvern, UK). Origin 9.0 (OriginLab Corporation, Northampton, MA, USA) was used for PL curve fitting and Design-Expert 10.0 (stat Ease Inc., Minneapolis, MN, USA) was carried out for statistical analysis. The fluorescence QY was calculated based on the comparative method [51,52,53] using the following equation:

(1) $$\mathrm{QY}=Q_{R}\;\frac{I}{I_{R}}\;\frac{OD_{R}}{OD}\;\frac{\eta^{2}}{\eta_{R^{2}}},$$

where (*I*) is integrated intensity, (*OD*) is optical density and (η) is the refractive index. Quinine sulphate in 0.1 M H_2_SO_4_ (QY=54%) was used as a fluorophore reference.

### 2.4. Procedures for Cu^2+^ Sensing

In a typical assay, the fluorescent probe was prepared as follow: 75 μM of the as-made N–CDs were taken in a 50 mL measuring flask, filled up to the mark using DI, mixed completely and used throughout all the detection experiments. The sensitivity of the N–CDs-based sensor towards Cu^2+^ ions was performed by adding 2 mL of probe into 2 mL of different concentrations of Cu^2+^. After incubation for 1 min at room temperature, the PL spectra were recorded with excitation/emission peaks at 350/465.5 nm. The selectivity study was assessed by adding 50 µM of metal cations, including Fe (III), Mg (II), Fe (II), Pb (II), Ca (II), Cu (II), Ni (II), Cd (II), Hg (II), Zn (II), Ag (I), Co (II), Li (I), Ba (II), K (I), Al (III) and Mn (II) ions into the probe using the aforementioned procedure.

### 2.5. Analysis of Real Samples

The water sample was acquired from the tap of biochemical lab (UPM, Selangor, Malaysia). Prior to the detection, the sample was filtered using a 0.22 μM filter membrane. The samples were then spiked with different concentrations (15, 30, 60 μM) of the copper solution and analysed using the developed sensing system. The precision of the analysis technique was evaluated based on the relative standard deviations (RSD) using three replicate measurements according to the following formula:

(2) $$\mathrm{Recovery}\;(\%)=S_{\mathrm{Spiked}}\times R_{\mathrm{Real}}/S_{\mathrm{Spiked}}\times 100\%,$$

where $S_{\mathrm{Spiked}}$ = calculated Cu (II) in spiked sample (µM) and $R_{\mathrm{Real}}$ = Cu (II) in the real sample solution (µM).

### 2.6. Immobilization of PVA/N–CDs Composite Film

The preparation of the PVA solution was done by having 1 g of PVA powder dissolved into 10 mL of DI water. Specifically, the mixture was heated at 90 °C and constantly stirred for 2 h until the powder was completely dissolved. After that, 1 mL of 1 mg mL^−1^ N–CDs was mixed with 1 mL of PVA solution and stirred continuously. Once the homogenous gel mixture was formed, it was poured into a clean glass substrate. The heating process for this mixture was heated in an oven at 80 °C for 1 h. Finally, the PVA/N–CDs composite film was peeled off from the glass slide surface so that a freestanding film could be obtained.

## 3. Results and Discussion

### 3.1. Synthesis of N–CDs

**Scheme 1:**
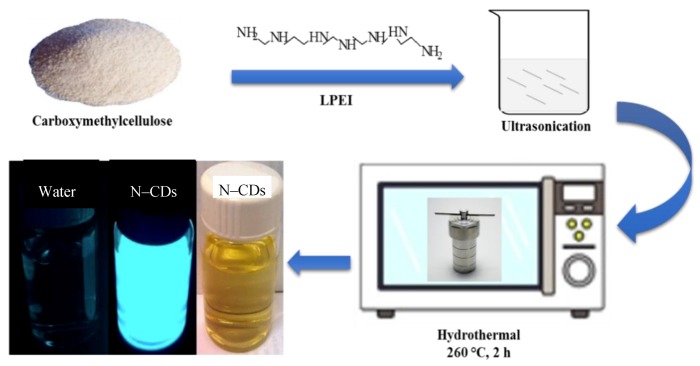
Schematic representation of the preparation of fluorescent N-doped carbon dots (N–CDs) from carboxymethylcellulose (CMC) and linear-structured polyethyleneimines (LPEI).

The fluorescent N–CDs were prepared from CMC of oil palm empty fruit bunch with the incorporation of LPEI using a facile hydrothermal carbonisation (HTC) route. The synthesis preparation of the N–CDs is illustrated in Scheme 1. EFB cellulose mainly comprised of D-glucose subunits, which were linked to each other by β-(1,4)-glycosidic bonds. The abundance of hydroxyl and ether groups within the CMC structure could provide the elemental and structural basis for the formation of CDs [53]. Since CMC does not contain N-functionalities in its framework, obtaining superior PL properties of these renewable bioresources is the critical step. The abundant primary and secondary amino groups in the backbone basis of LPEI polymer could play three basic roles, which are (i) a surface passivation species; (ii) a N incorporation for obtaining N–CDs; and (iii) cupric amine formation to increase the coordination interaction of N–CDs with metal ions.

To obtain the optimal fluorescent efficiency of N–CDs, several variables, including synthesis temperature, reaction time, and dosage of LPEI were optimized, as seen in Tables S2 and S3. Under optimal conditions, N–CDs with QY of up to 44% were obtained. The negative effect of N dosage on QY (Table S2) could be related to the wrapping effect of N–CDs with excessive amino groups, leading to the reduction of surface defects and result in lower QY [18,54]. Moreover, two hours of synthesis duration was found to be sufficient for efficient carbonisation conversion rate and obtaining highest QY, in which a further increase in time could result in the decomposition of reactive functional species during the HTC process (Table S3). However, it is believed that only a polymerization reaction might have occurred as a result of the short reaction time. It is worth mentioning that short synthesis time of the present study was sufficient for obtaining higher QY of N–CDs compared to CDs synthesized via HTC treatment of different natural and waste materials (Table S1).

### 3.2. Surface Morphology and Structural Analysis of N–CDs

**Figure 1:**
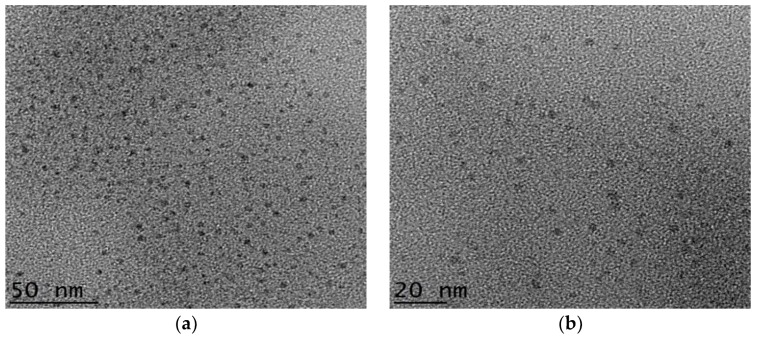

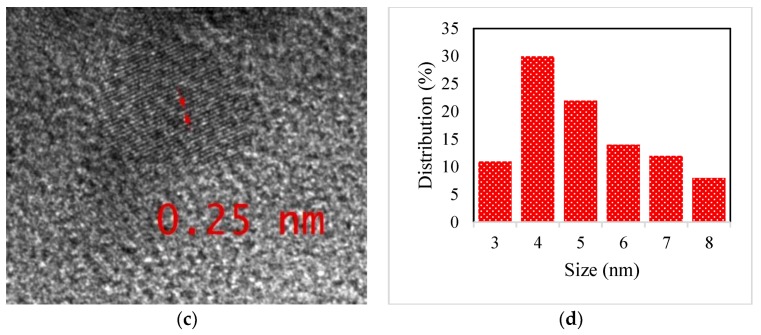
TEM images of the N–CDs in different magnifications (**a**,**b**), high-resolution (HR)TEM image (**c**) and the histogram showing the distribution particle size (**d**).

The surface morphology of N–CDs was characterised by TEM and HRTEM. As shown in Figure 1a,b, N–CDs were homogeneous, with quasi-spherical and monodispersed particles. According to the particle size distribution histogram, which was calculated from one hundred particles (Figure 1d), the N–CDs had a size distribution ranging from 3 to 8 nm with an average size of around 3.4 ± 1 nm. The HRTEM image (Figure 1c) revealed that most particles exhibited a clear crystalline structure with an interplanar distance of 0.25 nm. This distance was near to the value for the [002] planes of a graphitic-like structure. The crystallinity lattice framework of the N–CDs, which was observed in the HRTEM images suggested that these nanodots had the inner core of graphite. This finding was in agreement with those reported in previous studies [55,56,57].

**Figure 2:**
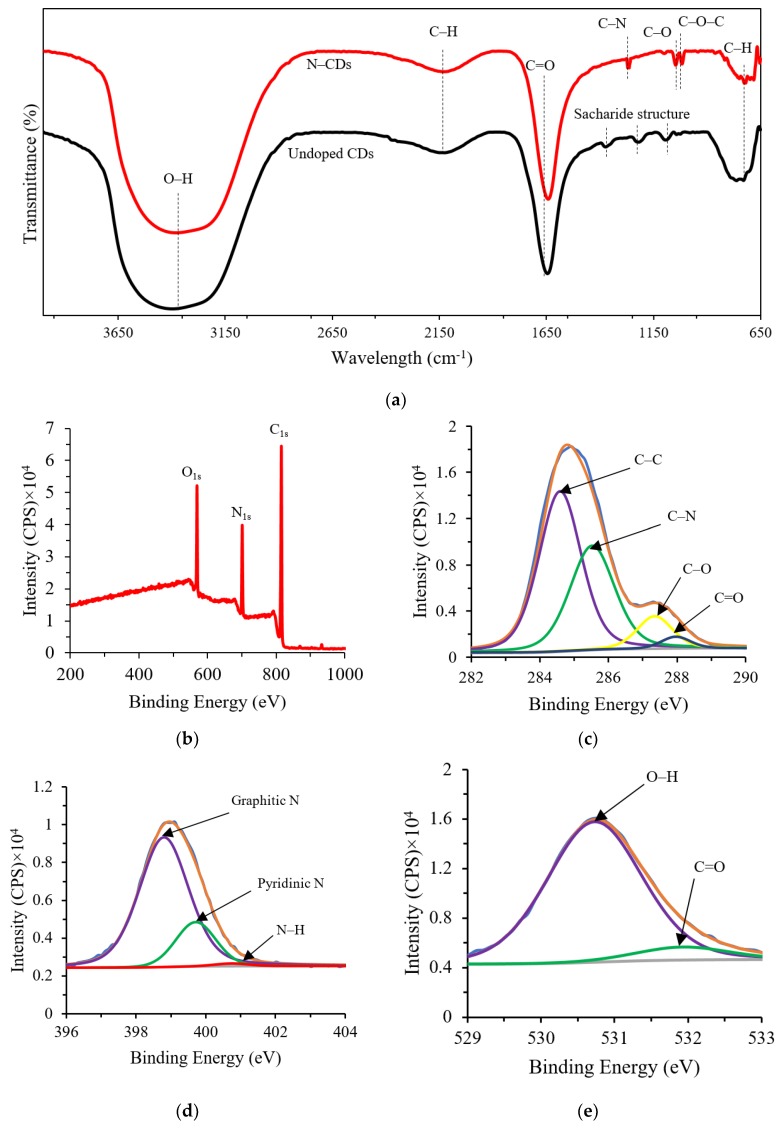
(**a**) FTIR analysis of undoped (black line) and N–CDs (red line), (**b**) X-ray photoelectron spectrum (XPS) of N–CDs, (**c**) high-resolution XPS for C_1s_, (**d**) high-resolution XPS spectra for N_1s_ and (**e**) high-resolution XPS spectra for O_1s_.

The FTIR spectrum shown in Figure 2a presents typical absorptions of undoped and N–CD aqueous solutions. It could be seen from the graph that there was a sharing absorption peak at 3381.43 cm^−1^, which corresponded to the O–H stretching vibration. Meanwhile, the peaks at 2124.54 cm^−1^ and 725.11 cm^−1^ assigned to C–H stretching and bending vibrations, respectively [20,58,59]. In comparison to the C–H bending vibrations of N–CDs, the peak of undoped CDs was wider. The existence of these hydrophobic species could be correlated with the PL loss due to decreased carrier mobility in the polar environment [60]. The stretching vibration centred at 1641.26 cm^−1^ might have been attributed to C=O groups, suggesting the formation of carboxylic acid groups [29]. Moreover, the bands at 1373.44, 1218.04 and 1093.77 cm^−1^ corresponded to the saccharide structure [56].

The presence of LPEI in the carbon framework of N–CDs could lead to significant changes compared to the undoped CDs. More specifically, the peak of C–N at 1266 cm^−1^ was formed after LPEI addition. This peak could have resulted from the destruction of the CMC saccharide structure and loss of characteristic vibrations via dehydration and passivation process [54,56]. Meanwhile, the bending vibration of C–O and C–O–C appeared at wavenumber of 1045.87 and 1016.57 cm^−1^, respectively, indicating that oxidized species were present [7,29]. The above investigations confirmed that hydroxyl, carboxyl, carbonyl and amino containing groups were formed through the HTC of CMC and LPEI, which imparted hydrophilicity and stability to N–CDs via creating the energy gaps [5].

To investigate the surface chemical composition of N–CDs, both the undoped and N–CDs were characterized using Energy-dispersive X-ray spectroscopy (EDS) as presented in Table S4. In comparison to undoped CDs, the carbon content of N–CDs was greatly increased from ~54.3% to ~64.6% along with the decrease in the contents of oxygen. This indicated the high level of aromatization with the elimination of oxygen functional groups. Additionally, the presence of nitrogen contents with atomic ratio of ~19.4% after doping CDs with LPEI confirmed the successful synthesis and embedment of amino species into the final surface structure of N–CDs [61].

XPS spectra was applied to verify the surface composition of the N–CDs. As shown in Figure 2b, the as-synthesized N–CDs exhibited three peaks, which mainly corresponded to carbon, nitrogen and oxygen (C: 64.61 wt%, N: 19.38 wt%, O: 11.23 wt%). The nitrogen weight content of the present N–CDs is much higher than the N–CDs obtained from nitrogen-doped CDs using different natural carbon sources such as peach gum (14.1%) [62], Actinidia deliciosa (6.9%) [61] and grass (4.23%) [54]. The spectrum of the C_1s_ region (Figure 2c) was deconvoluted into four single peaks attributed to various C states. The dominant peak at 284.59 eV was ascribed to sp^3^-hybridized (C–C) carbon, while the other peaks at 285.53, 287.35 and 287.97 eV corresponded to C–N, C–O and C=O bonds, respectively. Based on the N_1s_ spectrum shown in Figure 2d, three peaks of graphitic N–(C)_3_, pyridinic N and N–H were observed at 398.8, 399.7 and 400.75 eV, respectively. This observation gave evidence that nitrogen had been effectively doped into the sp^2^-conjugated domains of N–CDs in different modes [18,20], which was consistent with FTIR measurement results. The O_1s_ spectrum shown in Figure 2e confirmed the existence of two characteristic oxygen states of O–H and C=O at 530.73 and 531.93 eV, respectively. The XPS data suggested that the obtained N–CDs were greatly functionalized by different polar oxygen/nitrogen groups. These findings were in agreement with the results obtained from FTIR and EDS analysis.

### 3.3. Optical Properties of N–CDs

**Figure 3:**
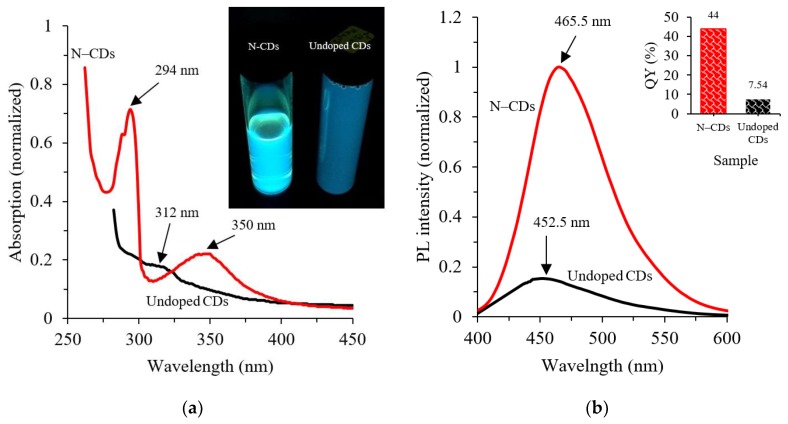

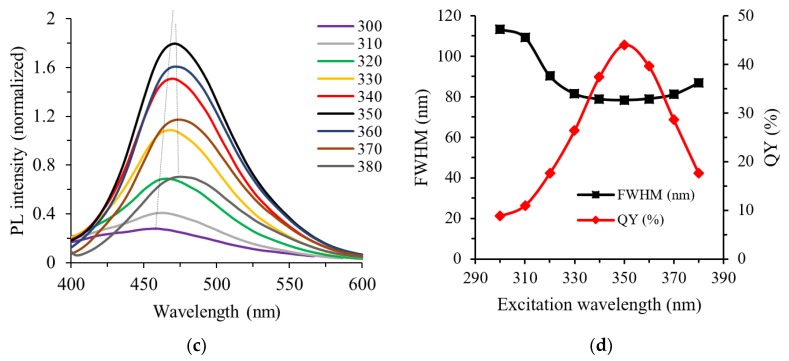
(**a**) Absorption spectra of undoped and N–CDs, (**b**) fluorescence PL spectra of undoped and N–CDs excited at 350 nm, with A_350_ = 0.04, (**c**) PL spectra of N–CDs under various excitation wavelengths (300–380 nm) and (**d**) emission full width at half maximum (FWHM) and quantum yield (QY) as a function of excitation wavelength. The inset in panels (**a**) for the corresponding photographs under 365 nm UV light and (**b**) the corresponding calculated QYs.

The UV-vis absorption spectra of the undoped CDs and N–CDs are presented in Figure 3a. It is shown that both curves exhibited long absorption edge in both UV and visible region (300–600 nm) without any noticeable sharp absorption peaks, as mostly observed in CDs [63]. A typical absorption peak at 294 nm was observed in N–CDs, which was assigned to the π-π* transition of sp^2^-hybridized carbon core (C–C) [63]. Besides that, peaks were observed at 312 and 350 nm in undoped CDs and N–CDs, respectively, which may have been attributed to n-π* of non-conjugated electron orbitals, originated from the oxygen functionalization (C=O) bonds and amino groups at the surface (in case of N–CDs) similar to previous studies [21].

The colour of the well-dispersed CDs and N–CDs (inset of Figure 3a) exhibited blue and greenish emission, respectively while irradiated with 365 nm ultraviolet (UV) light, indicating the dependence of the PL emission on the particle sizes and the sp^2^-carbon framework. It was also proven that both larger conjugated system and the higher graphitization of nitrogen-doped polyaromatic structure played a main role in reducing the energy bandgap, and hence results in red-shifted of PL emission [3,20,64]. The strong greenish photoluminescence of N–CDs can be clearly noticed with the naked eye, making them promising applications in a wide range of areas, such as bioimaging, biomedical, and chemical sensing.

The PL spectra of undoped and N–CDs solutions excited at 350 nm is presented in Figure 3b. Based on the graph, it can be seen that N–CDs had a stronger emission compared to the undoped CDs with the same absorbance. Besides, there was red-shift of the PL maxima of N–CDs. The inset of Figure 3b shows that the maximum PL QY of N–CDs in aqueous solution was approximately 44%. This efficiency was higher than the maximum fluorescence QY of the undoped CDs, which was 7.54%. Meanwhile, it was proven in this study that LPEI was non-emissive when it was hydrothermally synthesized on its own. This indicated the significant role of using LPEI as surface passivating agent/nitrogen source for the enhancement of PL properties of CDs.

The selective luminescence spectrum of N–CDs is illustrated in Figure 3c. When excited by light with different excitation values (300–380 nm), wide emission features could be observed, in which the excitation increased to a maximum of 350 nm and then reduced rapidly afterwards. Blue-shift of emission peaks was shown when the excitation wavelength was below 350 nm. However, red-shift was present at 472.5 when excitation wavelength varied from 350 to 380 nm. This excitation dependence/independence emission wavelength was most likely due to the surface state emission originated from the abundant of reactive oxygen/nitrogen functional groups, which affects the energy bandgap of N–CDs [18,65]. Additionally, the selective luminescence excitation wavelength was also possibly due to the size variation of sp^2^-hybridized carbon framework and the graphitic nitrogen content [56,64,65].

Figure 3d shows the full width at half maximum (FWHM) of the emission spectra as a function of excitation wavelength. A slight change of excitation wavelength in the range of 320–380 nm were observed with a minimum FWHM of 78.3 nm at λ_ex_ = 350 nm. Meanwhile, a gradual increase in the FWHM could be seen when the excitation value was lower than 320 nm. This suggested the suitability of using these nanomaterials in broad range of analytical applications [66]. Moreover, Figure 3d shows that PL QY was highly dependent on the excitation wavelength. Specifically, the rise in the QY occurred with the increase of excitation wavelength, reaching the maximum of ~44% at 350 nm excitation. However, further increase of excitation wavelength had a negative effect on the fluorescence QY. Comparing the dependence of QY and FWHM indicated a trade-off between QY and FWHM. There was a similarity between the peak of absorption and excitation wavelength, which corresponded to the maximum intensity of emission at 350 nm. This similarity demonstrated that non-emitting absorption arised in N–CDs. The N–CDs had a large Stokes shift of around 115.5 nm, which is an advantage for bio-imaging applications [3].

The formation of N–CDs, which was investigated in this study, took place via five pathways: dehydration, polymerization, aromatization, nucleation and growth processes [55,67]. A schematic representation of the fundamental understanding of the N-CDs formation from CMC and LPEI through HTC is shown in Figure S1. More details on the proposed chemical reactions for the formation steps of N–CDs can be found in the Supplementary Materials.

### 3.4. Effect of N–CDs Concentration, pH, Surface Charge, Irradiation Time, Ionic Strength and Storage Time on the Fluorescence of N–CDs

**Figure 4:**
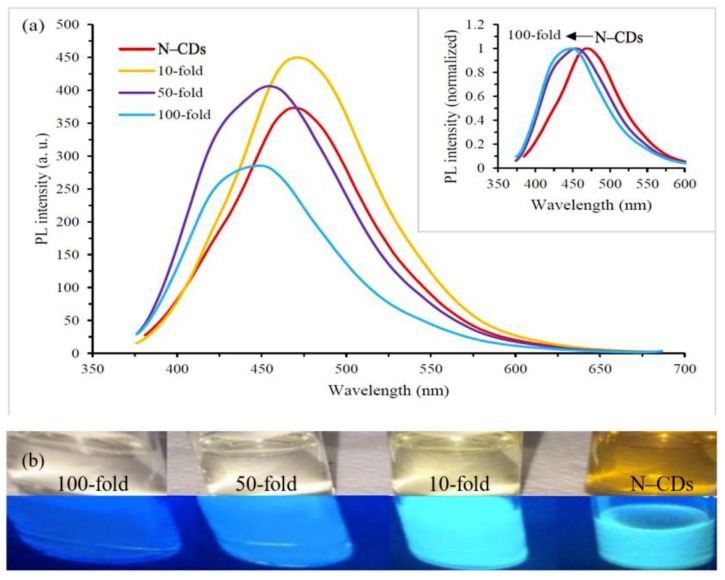
PL spectra of the N–CDs at different dilution factors starting with N–CDs, 10, 50 and 100-fold (**a**) and the corresponding digital images at different dilutions under daylight (above) and UV-light (365 nm) (below) (**b**). Inset shows the normalized fluorescence emission spectra of N–CDs at different dilutions with the excitation wavelength of 350 nm.

Despite the superior optoelectronic properties of N–CDs, there are insufficient studies on the intensity wavelength and PL property as a function of N–CDs concentration, in which PL is usually measured in most works of literature without any dilution. Prior to applying N–CDs for further applications, several dilutions (10, 50 and 100-fold) of the originally obtained N–CDs were carried out, as shown in Figure 4. It could be seen that the intensity wavelength and the PL emission of the N–CDs were highly dependent on the concentration of N–CDs solution. As shown in Figure 4a, the emission peaks were constant before and after the addition of 10-fold dilution factor, with emission maxima of 465.5 nm. However, gradual blue-shift in the emission maxima (from 465.5 to 448 nm) were observed with continuous 50 and 100-fold dilution with DI water. This blue-shift could be clearly seen in the normalized fluorescence emission spectra (inset of Figure 4), in which different dilutions were excited under a similar wavelength value of 350 nm.

A progressive rise in the PL intensity of the emission peak could be seen with the reduced N–CDs concentration, as shown in Figure 4a. The corresponding images of N–CDs without and with DI dilution (10, 50 and 100-fold) under daylight and UV-light, respectively are shown in Figure 4b. The colour of aqueous N–CDs under ambient light gradually changed from dark yellowish to transparent with the increased addition of DI water into the obtained N–CDs. The results regarding the irradiation of the samples by the UV-light (λ_ex_ = 365 nm) was in agreement with the results of PL spectra, in which the addition of DI water dilution by 10-fold led to the highest PL emission, in comparison to the concentrated N–CDs.

The variation of emission phenomena could be explained by the combination of Van der Waals forces to create nano-sized clusters at a high-concentration solution. This would lead to an increased polarity on the surfaces of aggregated N–CDs. However, with the addition of DI water of up to 10-fold dilution into the N–CD solution, these aggregated N–CDs were separated into monodispersed N–CDs. As a result, the polarity and the emission enhancement of N–CDs would be reduced [2]. Overall, it was suggested from the results that the adjustment of aqueous N–CDs concentration was the key point to tune the emission wavelength and fix the emission peak, in which the high polarity nanoclusters may lead to diverse emission phenomena [68].

**Figure 5:**
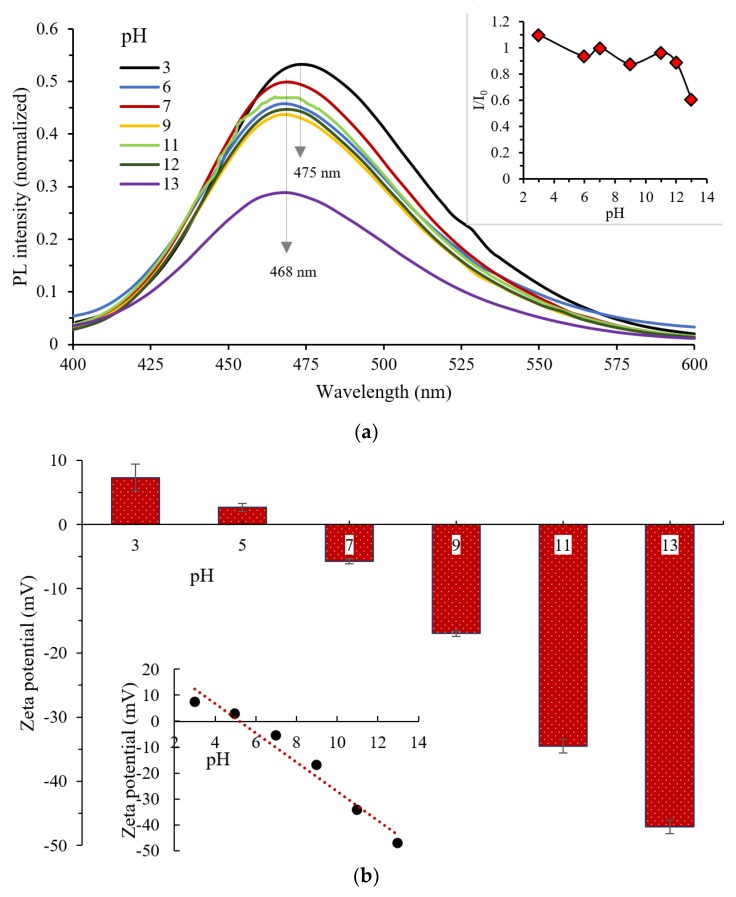
(**a**) The emission dependence of N–CDs on pH values with λ_ex_ = 350 nm and (**b**) zeta potential study of N–CDs at various pH values. Inset: (**a**) the effect of different pH values on the relative intensity and (**b**) the linear relationship between pH and zeta potential.

The PL of the numerically optimized N–CDs was further studied by identifying the influence of pH, surface charge, time of exposure, ionic strength and storage time on the PL of N–CDs. The pH value of the original aqueous dispersion of N–CDs was around 7.6. The influence of pH level on the PL of N–CDs is shown in Figure 5a. PL emission at different pH values was analysed by adjusting aqueous N–CDs with NaOH and HCl. It can be said that when pH is about 3, the highest PL emission intensity could be reached. However, the PL emission would then weaken beyond this value until the PL emission turned to the lowest value under pH > 12. It was also observed that the emission was almost maintained completely with the pH value ranging from 6 to 12 (inset of Figure 5a). This proved the stability of the obtained N–CDs under a wide range of pH values. Moreover, red-shift in the emission maxima from 468 nm to 475 nm was observed at pH = 3. However, the emission maxima were constant under other pH conditions. This phenomenon could be explained by the surface state, in which various hydrophilic containing groups like –OH and –COOH are subjected to deprotonation at pH > 12. This process would form anions which could change the PL position and intensity due to the N–CDs surrounded by anion aggregate [20,69,70].

The pH mechanism of N–CDs was further evaluated by zeta potentials (ξ) study. Based on the results shown in Figure 5b, ξ potential changes from 7 mV to −47 mV with the increase of pH value from 3 to 13. A linear relationship (inset of Figure 5b) between ξ potential and pH (3–13) could be expressed as ξ = −5.6436pH + 29.432 (*R*^2^ = 0.9654). The high ξ potential confirmed the reversible protonation and deprotonation of the reactive containing groups, especially –NH and –COOH, on the surface of N–CDs which facilitated the negatively-charged zeta. As a result, the electrostatic interactions between the N–CDs and negatively-charged surface could prevent the aggregation of the N–CDs and hence stabilize them [71]. ξ potential measurement indicated that the obtained N–CDs had an isoelectric point at pH 5.7. The overall results of ξ potential study support very well the data obtained from FTIR and XPS analysis. It is worth mentioning that the ξ potential of the aqueous N–CDs of the present work (−47 mV) was higher than that of the CDs obtained using different passivation agents such as xylan/NH_4_OH (−4.26 mV) [72] and S–CDs from thiomalic acid/H_2_SO_4_ (−42 mV) [73]. This indicated the highly negatively-charged groups presented over the N–CDs surface which imparted higher stability and dispersibility.

The characteristics of PL intensity and the UV exposure time up to 180 min is presented in Figure S2a. The PL intensity exhibited no significant decrease after 180 min of illumination, retaining about 97% of their original PL emission. This suggested that the N–CDs were highly resistant to photobleaching. Similar to the effect of UV exposure time, the NaCl and KCl concentrations showed no obvious effect on the PL emission even when the concentration of NaCl and KCl was up to 1.0 M (Figure S2b,c). This demonstrated the excellent stability of the LPEI–CDs under high-salt conditions. Moreover, with the rise of temperature from 25 °C to 60 °C (Figure S2d), PL intensity retention of around 97.8% was observed. The effect of storage time on the PL intensity of N–CDs was also investigated. As shown in Figure S3a, the obtained N–CDs were stable for more than six months without any aggregation. The residual PL intensity of the N–CDs retained 95.4% of their original value after six months (Figure S3b). The outstanding photostability of N–CDs was possibly due to their small size effect, graphitic nitrogen content and the reactive containing groups which were formed on the N–CDs surface. The long shelf-life of N–CDs suggested that there is a possibility of applying these N–CDs for commercial applications, such as biological labelling and fluorescent chemical sensing.

### 3.5. Fluorescence Quenching for Cu^2+^ Sensing

**Figure 6:**
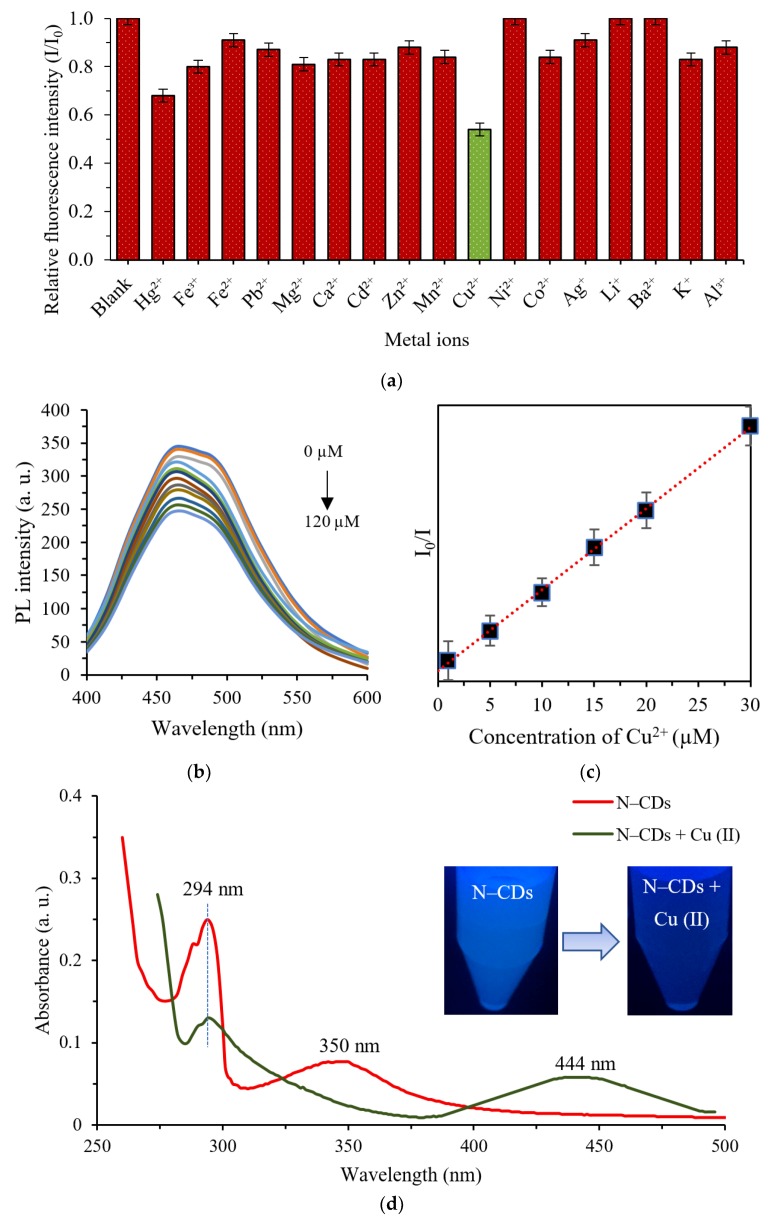
(**a**) Relative fluorescence intensity of aqueous N–CDs at λ_em_ = 465.5 nm in the presence of 50 µM of various individual metal ions, (**b**) PL emission spectra of aqueous N–CDs in the presence of different concentration of Cu^2+^ (0, 15, 30, 50, 70, 90 and 120 µM), (**c**) a linear correlation of I_0_/I ratio and the concentration of Cu^2+^, where I_0_ and I represent the fluorescence intensity in the absence and in the presence of Cu^2+^ ion, respectively and (**d**) UV-Vis absorption spectra of N–CDs before (red line) and after (black line) the addition of 700 μM Cu (II). Inset shows the corresponding photo images of sensing under UV lamp.

FTIR and XPS studies confirmed the existence of oxygen/nitrogen-containing groups over the N–CDs surface. These groups have good affinity to interact with metal ions by forming coordination bonds, in which donation of electron pair from the functional terminal to the centre metal ions can occur. Hence, the selectivity of the obtained N–CDs towards the most commonly found metal ions in water system was examined as presented in Figure 6a. *I* and *I*_0_ represent the PL intensities of the probes at 465.5 nm in the absence and presence of 50 µM of various metal cations, respectively. It is shown that the addition of Cu^2+^ caused a drastic reduction in the PL emission of N–CDs with over 50% of quenching efficiency, while less was noticed by adding other metal ions. The above results indicated that only Cu^2+^ had higher chelating kinetics with N–CDs owing to the higher binding of Cu^2+^ to N–CDs at the –OH, –COO^−^, and/or –NH, resulting in the fluorescence quenching effect [74].

According to the response selectivity of CDs to Cu^2+^ among other metal ions, three factors may be attributed for this: (i) copper ions exhibit the highest absorption affinity towards the carbon nanostructure among other transition metals, which is the prerequisite for the effective fluorescence quenching for the CDs/Cu^2+^ system [75]; (ii) the strong fluorescence quenching for CDs/M*^n^*^+^ (M: metal) system means the efficient electron transfer from the excited state of CDs to M*^n^*^+^, then back to the ground state of CDs, which requires an appropriate potential of M^n+^/M (i.e., more negative than the potential of the hole on the CDs and more positive than that of the electron on the CDs). From the standard formal potential (*E*_0_) of M*^n^*^+^/M, the redox potential of Cu^2+^/Cu^+^ (or Cu^2+^/Cu) was possibly optimum for the electron transfer from CDs compared to other reference metal ions [76]; (iii) the good oxidisability of Cu^2+^ makes Cu^2+^ the most appropriate receptor for the transferred electron from QDs [77].

In order to achieve high analytical performances, several sensing conditions, including response time and pH values were analysed and optimized prior to sensitivity measurements. The effect of time durations (from 1 to 25 min) on the PL quenching of N–CDs is presented in Figure S4a. Highest quenching degree in the presence of 50 μM Cu^2+^ was observed within 1 min (λ_max_ = 350 nm). However, less to no effect was noticed upon increasing sensing time, indicating the rapid detection signal of LPEI–CDs towards Cu (II). A fast response time of 1 min, much faster than the reported ones [69,70,71,78], made the LPEI–CDs quick-detection sensors for Cu^2+^ in aqueous water.

The pH value of the N–CDs solution in the presence of Cu^2+^ was another key factor that affected the sensing system. Thus, the effect of pH values (ranging from 3–13) on the PL quenching of N–CDs was evaluated and the results are shown in Figure S4b. The PL response of N–CDs to Cu^2+^ is evidently weakened in strong acid or alkaline conditions, which should be due to the protonation of the carboxyl group under acidic conditions or generation of Cu(OH)_2_ precipitation under alkaline conditions, respectively [79]. Based on the above findings, the sensitivity experiments were performed under optimal conditions of 1 min incubation time at pH 7.

In order to investigate the sensitivity of the N–CDs as a probe for Cu (II) ions sensing, analytical characteristic study was conducted. The relative intensity at 465.5 nm was utilised as the sensing signal for the sensitivity study. The PL of N–CDs was quenched accordingly and dependently to the concentration of the Cu (II) added to the N–CDs. However, the degree of change in the signal intensity corresponding to the concentration of Cu (II) was found to be non-linear. The variation of PL intensity of the aqueous N–CDs in the presence of different concentration of Cu^2+^ is shown in Figure 6b. A gradual quenching in the PL intensity was shown with the rise of Cu (II) concentrations (from 0–120 µM). Based on the Stern–Volmer equation [80], the linear relationship between the relative PL intensity ratio (*I*_0_/*I*) versus the concentration of Cu (II) within the range of 1–30 µM was obtained (Figure 6c), with a correlation coefficient of *R*^2^ = 0.9994. The fluorescence quenching efficiency of the Cu (II) would be described through to the following equation:

(3) $$I_{0}/I=1.0476+0.0056\times C_{\mathrm{Cu}}^{2+},$$

where *I*_0_ and *I* are the fluorescence intensity in the absence and presence of Cu (II) ion, respectively. The detection limit (LOD) of Cu^2+^ (at a Signal/Noise of 3) was estimated to be 0.93 µM based on LOD =3S/K, where K refers to the calibration curve slope and S represents the standard deviation of the blank. Table 1 outlines the performance parameters of the recently reported work done for the sensing of Cu^2+^. A low detection limit of 5 nM and wide detection range of 5 nM–100 μM was obtained for CdSe QDs, but the introduction of toxic element inhibits their practical application [50], though it can be enhanced by cladding with a silica shell [76].

As a contrast, CDs are excellent fluorescence probes due to their non-toxicity, high stability and sensitivity, but some shortcomings inhibit their ion detection application, such as long response time of more than 10 min [77,81,82,83], narrow detection interval of only two orders of magnitude [82,84,85], high detection limit [80,83,86,87] or complex probe synthesis [83,85]. The complicated processing could be avoided during the synthesis of the N-doped CDs, but these N–CDs exhibited narrow detection intervals of 0.3–1.6 μM [87]. In comparison to a number of previous reports, the present nanoprobe had beneficial performance in terms of detection interval, response time and LOD as well as QY.

The maximum recommended level of Cu (II) ions in drinking water are 2 mg/L, equivalent to 47.2 μM and 1.3 mg/L, equivalent to 20.46 μM as suggested by the World Health Organization and Environmental Protection Agency, respectively [48]. The low LOD for Cu (II), which was obtained through the proposed sensing probe in this research, could be explored and applied for its potential in detection of Cu (II) in water samples.

### 3.6. Investigation of Sensing Mechanism

Based on the earlier observations [88,89,90], the high affinity of N–CDs by Cu^2+^ might be attributed to the cupric amine complex, which could be formed by the coordination of Cu^2+^ ions with active amino groups around N–CDs. PL intensity reduction might be resulted from this stable complex leading to N–CDs aggregation. To prove the aforementioned assumption, different spectroscopic measurements were carried out to study the characteristics of N–CDs in the presence of Cu (II).

As seen in Figure S5, there was a significant increase by ~600nm in the average size of N–CDs after Cu (II) was added. This indicated the aggregation of N–CDs chelated by Cu (II) through the formation of Cu (II)–N–CDs complexes. Furthermore, the zeta potential value increased from −7.74 to 20.97 eV after the addition of 700 μM Cu (II), as shown in Figure S6. This increase suggested that the negative charges of N–CDs were neutralized with the positive charges of Cu (II) due to the electrostatic effect. UV-Vis spectroscopy of N–CDs was also measured in the presence of 700 μM Cu (II) as shown in Figure 6d. It can be observed that the absorption of the N–CDs after the addition of Cu (II) exhibited a new band at 444 nm and the absorption peak at 350 nm totally disappeared. This additional band could be ascribed to the charge transfer from N atom to the d orbitals of Cu (II) since the higher absorption energy peak, which was originated from sp^2^-hybridized carbon core (C=C), was unaltered. Moreover, the fluorescence reduction of ~50% supported the assumption that the quenching mechanism was caused by the binding effect of N species and not differently sized LPEI–CDs as its reduction was not fully quenched [87]. These above results confirmed that Cu (II) could form coordinate covalent bonds with the active organic N sites that fully covered the surface of LPEI–CDs, which enabled the design of “fluorescence turn-off” sensors for the rapid determination of Cu (II).

### 3.7. Analysis of Water Samples

In order to assess the practicality of the developed sensing probe, the detection of Cu (II) was investigated in tap water using a standard addition method. The experimental results of the blank and spiked real water samples before and after the addition of Cu (II), respectively are presented in Table 2. It is shown that there was no Cu (II) found in the blank sample. The results of spiked samples showed that the percentage of recoveries were between 97.03% and 105.82% and the RSD were from 1.49% to 2.43%, which was satisfactory for the application. In comparison to the previous works [74,91], our present sensing probe was of great advantage not only in terms of highly QY that were obtained using mild conditions but also of being less toxic compared to metal-based QDs. This suggests the reliability of applying the developed N–CDs as probes for the determination of Cu (II) in real water.

### 3.8. Composite Film

Up to now, exploring N–CDs for novel applications is still a hot topic area due to their fascinating properties like fluorescent long-shelf time, resistance to photobleaching and great transparency in the visible light. However, the immobilization of N–CDs in solid-state cases is still challenging since N–CDs suffers from self-quenching property after the fabrication process. It is well known that superior solid-state PL emission of N–CDs is of great advantage for light-emitting diode (LED) fields. Thus, a polymer matrix was developed using PVA/N–CDs composite film to evaluate the workability of non-ionic polymer to bind colloidal particles onto a solid surface. PVA was chosen as the polymer host because it is a non-toxic, hydrophilic, optical transparency, bioadhesive polymer [92,93].

**Figure 7:**
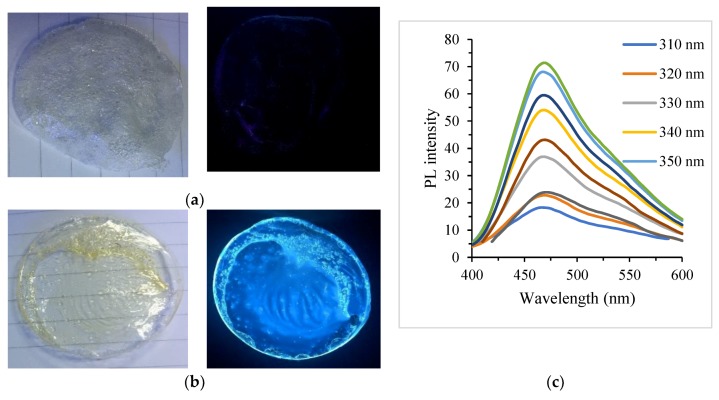
Photographs of poly (vinyl alcohol (PVA) film (**a**) before and (**b**) after encapsulating with N–CDs under ambient light (up) and UV light (down) and (**c**) PL spectra of PVA/N–CDs film at different excitation wavelengths (310–390 nm).

As shown in Figure 7a,b, the PVA film without the immobilization of N–CDs had no PL emission while PVA/N–CDs films exhibited a bright greenish PL under UV-light irradiation. This indicated that the N–CDs were properly encapsulated in the PVA film. The presence of the active hydrogen-containing groups (–COOH, –OH and –NH) on the N–CDs surface could form cross-linking chains. This led to the hydrogen bond formation from their plenty interactions with PVA [92,94]. In contrast to the aqueous N–CDs solution shown in Figure 3c, only an excitation independent feature was observed from the solid film upon various excitation wavelengths (Figure 7c), which is consistent with previous finding [95]. Additionally, the excitation/emission maxima of PVA/N–CDs film was red-shifted by 10/3 nm, respectively (Figure S7). This phenomenon may belong to PVA environment, which was a different case compared to the aqueous solution [96].

FTIR spectra were conducted in order to investigate the changes in the structure and surface functionalities between the obtained N–CDs solution and PVA/N–CDs solid film, as presented in Figure S8. In contrast to N–CDs solution, significant changes were observed after immobilization of N–CDs solution into the PVA host. To be specific, new peaks at 1438.25 and 1738.43 cm^−1^ were ascribed to C=C and C=N groups, respectively [95]. Meanwhile, the shifting and rise of C–N from 1266 to 1368 cm^−1^, implied an increase in graphitic structure after the encapsulation process. Furthermore, the change in position and shape of the C–H bending of N–CDs solution from 725.11 to 771.76 cm^−1^ confirmed the formation of strong hydrogen bonds between N–CDs and PVA chains [97]. Moreover, an obvious increasing and shifting of C–O and C–O–C species from 1045.87 to 1095.56 cm^−1^ and 1016.56 to 979.05 cm^−1^, respectively were noticed, which indicated that both the oxygen-related surface states and topological defects were increased [98]. Additionally, the peak of C=O stretching vibrations at 1641.26 cm^−1^ of N–CDs shifted to 1646.84, suggesting the formation of hydrogen bonding between the carboxyl groups of N–CDs surface and the hydroxyl groups of PVA [94]. All the changes in the peak positions and shapes confirmed the sufficient interaction between PVA and N–CDs.

The PL QY of the developed PVA/N–CDs film was calculated (Table S6) based on a comparative study and found to be 47%, which was higher than that of the N–CDs solution. The PL enhancement might be due to the strong hydrogen bonding formation, which provides more efficient radiative recombination of the electrons/hole (e–h) pairs [96]. Furthermore, the photostability of the PVA/N–CDs film was found to be stable and no significant PL reduction was noticed even after 72 h of continuous irradiation to UV-excitation source (Figure S9). The PL efficiency enhancement and photostability feature of the developed PVA/N–CDs film confirms the potential of applying these composite films for a wide range of fields including solid-state lighting systems.

## 4. Conclusions

It can be concluded that highly luminescence, eco-friendly and water-soluble N–CDs have been successfully synthesized from CMC with the incorporation of LPEI as surface passivation agent/N-doping source through a one-step HTC route. By investigating the effects of reaction temperature, time and LPEI dosage on the optical properties of N–CDs, the optimal synthesis conditions were 260 °C reaction temperature, 2-h duration and 1% of LPEI dosage. Under these optimal conditions, high PL QY of up to 44% was obtained, which was six times higher than undoped CDs under similar conditions. A bright greenish colour with excitation-dependent/independent emission was observed from the obtained N–CDs solution. More impressively, with the reduced concentration of the obtained N–CDs, PL emission enhancement and blue-shift in emission maxima were noticed. The highly photostable emission nature of N–CDs was explored for multiple applications. The as-made N–CDs could be significantly quenched by Cu^2+^ and a good relationship existed between the PL quenching ratio and the concentration of Cu^2+^, with LOD of 0.93 µM. Based on the vast information, coordination complexes could be formed between Cu^2+^ and nitrogen-containing species around N–CDs, leading to electron transfer and PL quenching. A solid-state composite film of PVA/N–CDs was developed and found to be highly transparent, stable and displayed only excitation independent emission behaviour. The PL QY of the PVA/N–CDs film was calculated to be 47%, which was higher than that of N–CDs solution. This PL enhancement could be assigned to the radiative recombination of the e–h pairs resulting from the strong hydrogen bonding formation. It is believed that the development of N–CDs composite film would have many potentials in fields of chemistry and biology in the near future.

## Figures and Tables

**Table 1:** Summary of some fluorescent probes for Cu^2+^ sensing.

Probe	Response Time (min)	Response Interval (μM)	LOD (μM)	Reference
CDSe	5	0.005–100	0.005	[50]
CdSe/Zns@CDs	—	1–100	1	[77]
CDs from sago waste	—	0–47	7.78	[76]
CDs from grass	10	5 × 10^−5^–50	0.001	[82]
CDs from peanut shell	10	0–5	4.8	[83]
CDs from MS	15	0–10	0.23 × 10^−3^	[81]
CDs from CA	—	0.37–2.5	0.63	[85]
N–CDs from EDA/P_2_O_5_	—	10–400	10	[87]
N–CDs from D-glucose/EDA	10	10–100	1.8	[84]
N–CDs from CA/NaBH_4_/PEI	<1	0–80	5.3	[79]
N–CDs from CA/PEI	—	0.3–1.6	0.12	[80]
N–CDs from CMC/LPEI	1	1–30	0.9	This work

Note: MS: mesoporous silica, CA: citric acid, P_2_O_5_: phosphorus pentoxide, EDA: ethylenediamine, NaBH_4_: sodium borohydride, PEI: polyethyleneimine.

**Table 2:** Determination of Cu (II) in tap water using N–CDs.

Added (μM)	Found in Tap Water (μM)	Recovery (%)	RSD (*n* = 3, %)
0	ND *	—	—
15	16	105.82	2.43
30	30.14	99.07	0.87
50	49	97.03	1.49

* ND: not detected.

## Supplementary Materials

The following are available online at https://www.mdpi.com/2079-4991/9/10/1500/s1, Figure S1: Fundamental understanding of the N–CDs formation pathway, Figure S2: PL intensity as a function of (a) illumination time irradiated with UV light (365 nm) and (b) NaCl concentration, (c) KCl concentration and (d) temperatures, Figure S3: (a) Digital images showing N–CDs in ambient light and N–CDs and water under UV-lamp after six months at room temperature and (b) effect of storage time on the fluorescence intensity of the N–CDs (0–6 months) at 25 °C, Figure S4: The effect of (a) sensing time and (b) pH value on the relative fluorescence quenching of N–CDs before and after addition of 50 μM of copper ions, Figure S5: DLS size distribution of N–CDs after the addition of 700 μM Cu (II), Figure S6: Zeta potential of N–CDs after the addition of 700 μM Cu (II), Figure S7: PL spectra of N–CDs solution and N–CDs-PVA film showing (a) excitation and (b) emission maxima, Figure S8: FTIR spectra of the as-synthesized N–CDs and PVA/N–CDs film, Figure S9: Photostability of N–CDs-PVA composite film, Table S1: A summary of different starting materials used for synthesis of CDs, Table S2: Optimization of LPEI concentration for the production of N–CDs, Table S3: Optimization of synthesis conditions for the production of N–CDs, Table S4: Elemental compositions of the undoped and N–CDs, Table S5: Quantum yield calculation of PVA/N–CDs film.
