# Supplementary material for: Facile Synthesis of Nitrogen-Doped Carbon Dots from Lignocellulosic Waste
Source: Nanomaterials (Basel). 2019 Oct 22;9(10):1500. doi: 10.3390/nano9101500 (PMC6835739; doi:10.3390/nano9101500)
Supplement: Supplementary file 1 [file nanomaterials-09-01500-s001.pdf]

**Supplementary data**

# Facile Synthesis of Nitrogen-Doped Carbon Dots from Lignocellulosic Waste

**Mohammed Abdullah Issa [1], Zurina Z. Abidin [1,*], Shafreeza Sobri [1], Suraya Rashid [1], Mohd Adzir Mahdi [2], Nor Azowa Ibrahim [3] and Musa Y. Pudza [1]**

[1] Department of Chemical and Environmental Engineering, Faculty of Engineering, Universiti Putra Malaysia, 43400 UPM Serdang, Malaysia; mohbaghdadi1@yahoo.com (M.A.I.); shafreeza@upm.edu.my (S.S.); suraya_ar@upm.edu.my (S.R.); pudzamusa@gmail.com (M.Y.P.)

[2] Department of Computer & Communications Systems Engineering, Faculty of Engineering, Universiti Putra Malaysia, 43400 UPM Serdang, Malaysia; mam@upm.edu.my

[3] Department of Chemistry, Faculty of Science, Universiti Putra Malaysia, 43400 Serdang, Malaysia; norazwa@upm.edu.my

* Correspondence: zurina@upm.edu.my

## Table S1. A summary of different starting materials used for synthesis of CDs

| Starting material | Synthetic method | QY | Reference |
|---|---|---|---|
| Banana juice | Normal heating, 150 °C, 4 h | 8.95% | [1] |
| Sugar cane juice | Hydrothermal, 120 °C, 3 h | 5.76% | [2] |
| Apple juice | Hydrothermal, 150 °C, 12 h | 4.27% | [3] |
| Grape juice | Hydrothermal, 180 °C, 12 h | 13.5% | [4] |
| Coconut water | Microwave treatment | 2.8% | [5] |
| Papaya | Hydrothermal, 200 °C, 5 h | 18.98% | [6] |
| Lime, $NH_4HCO_3$ | Hydrothermal, 180 °C, 7 h | 39.62% | [7] |
| Cabbage | Hydrothermal, 140 °C, 5 h | 16.5% | [8] |
| Honey | Hydrothermal, 100 °C, 2 h | 19.8% | [9] |
| Milk | Hydrothermal, 180 °C, 12 h | 9.6% | [10] |
| Milk | Hydrothermal, 180 °C, 8 h | 7.55% | [11] |
| Naked oats | Pyrolysis, 400 °C, 2 h | 3.0% | [12] |
| Flour | Microwave, 180 °C, 20 min | 5.4% | [13] |
| Gelatin | Hydrothermal, 220 °C, 24 h | 31.6% | [14] |
| Peanut shells | Pyrolysis, 250 °C, 2 h | 9.91% | [15] |
| Pomelo peel | Hydrothermal, 200 °C, 3 h | 6.9% | [16] |
| Grass | Hydrothermal, 180 °C, 3 h | 6.2% | [13] |
| Plant soot | $HNO_3$ reflux | 0.72% | [17] |
| Egg membrane | Microwave | 14% | [18] |
| Orange peel | Hydrothermal, 180 °C, 12 h | 36% | [19] |
| Urine | Carbonization, 200 °C, 12 h | 5.3% | [20] |

| | | | |
|---|---|---|---|
| Dried shrimps | Hydrothermal, 170 °C, 12 h | 54% | [21] |
| Glucose, PEI | Hydrothermal, 150 °C, 12 h | 2.86% | [22] |
| Cellulose, $(NH_4)_2CO_3$ | Hydrothermal, 180 °C, 12 h | 7.6% | [23] |
| Glucose | Ultrasonic, 4 h | 7% | [24] |
| Cellulose, urea | Hydrothermal, 180 °C, 72 h | 21% | [25] |
| CMC, urea | Hydrothermal, 210 °C, 12 h | 18% | [26] |
| CMC, EDA | Hydrothermal, 270 °C, 6 h | 22.9% | [27] |
| Microcrystalline cellulose, EDA | Hydrothermal, 240 °C, 12 h | 51% | [28] |
| Folic acid | Hydrothermal, 180 °C, 2 h | 23% | [29] |
| Citric acid, urea | Microwave heating, 5 min | 14% | [35] |
| Branched PEI | Hydrothermal, 200 °C,10 h | 54.3% | [30] |
| PEG | Microwave, 900 W | 16% | [31] |
| Candle soot | $HNO_3$ oxidation | 1.9% | [32] |
| Activated carbon | $HNO_3$ oxidation | 1.6% | [33] |

**Table S2. Optimization of LPEI concentration for the production of N-CDs**

| **No.** | **Starting materials ratio** | | **Quantum yield (QY)** |
|---|---|---|---|
| | **Carboxymethylcellulose (g)** | **LPEI dosage (g)** | |
| 1 | 0.1 | 0.05 | 35.16 |
| 2 | 0.1 | 0.1 | 38.6 |
| 3 | 0.1 | 0.15 | 26.47 |

**Table S3. Optimization of synthesis conditions for the production of N-CDs**

| **No.** | **Synthesis temperature (°C)** | **Reaction time (hr)** | **Quantum yield (QY)** |
|---|---|---|---|
| 1 | 220 | 2 | 25.3 |
| 2 | 240 | 2 | 38.6 |
| 3 | 260 | 2 | 44 |
| 4 | 260 | 1 | 29.5 |
| 6 | 260 | 3 | 32.7 |

**Table S4. Elemental compositions of the undoped and N-CDs**

| Sample | C/atomic % | O/atomic% | N/atomic% |
|---|---|---|---|
| CDs | 54.3 | 32.6 | ND* |
| N-CDs | 64.6 | 11.2 | 19.4 |

Note. ND: not detected.

**Proposed chemical formation of as-prepared N-CDs**

Until now, there is no clear explanation to the possible chemical reactions occurring for the formation of N-CDs. However, the schematic representation of the possible formation mechanism of N-CDs is outlined in Figure S1. based on the vast findings characterized in this study and the literature reports of HTC of polysaccharides and N- doping source [34][35][36][37][38][39]. The mechanism shows that at long-time HTC process, decomposition of CMC into glucose monomer is occurred. At the same time, the amino species of LPEI can react with the aldehyde of glucose molecule to form glucosamines. Thus, hydroxymethylfurfural (HMF) is generated through multiple dehydration and fragmentation reactions of amino ketones or Amadori compounds [40]. Additionally, HMF could be formed through the decomposition products of vitamin C [41].

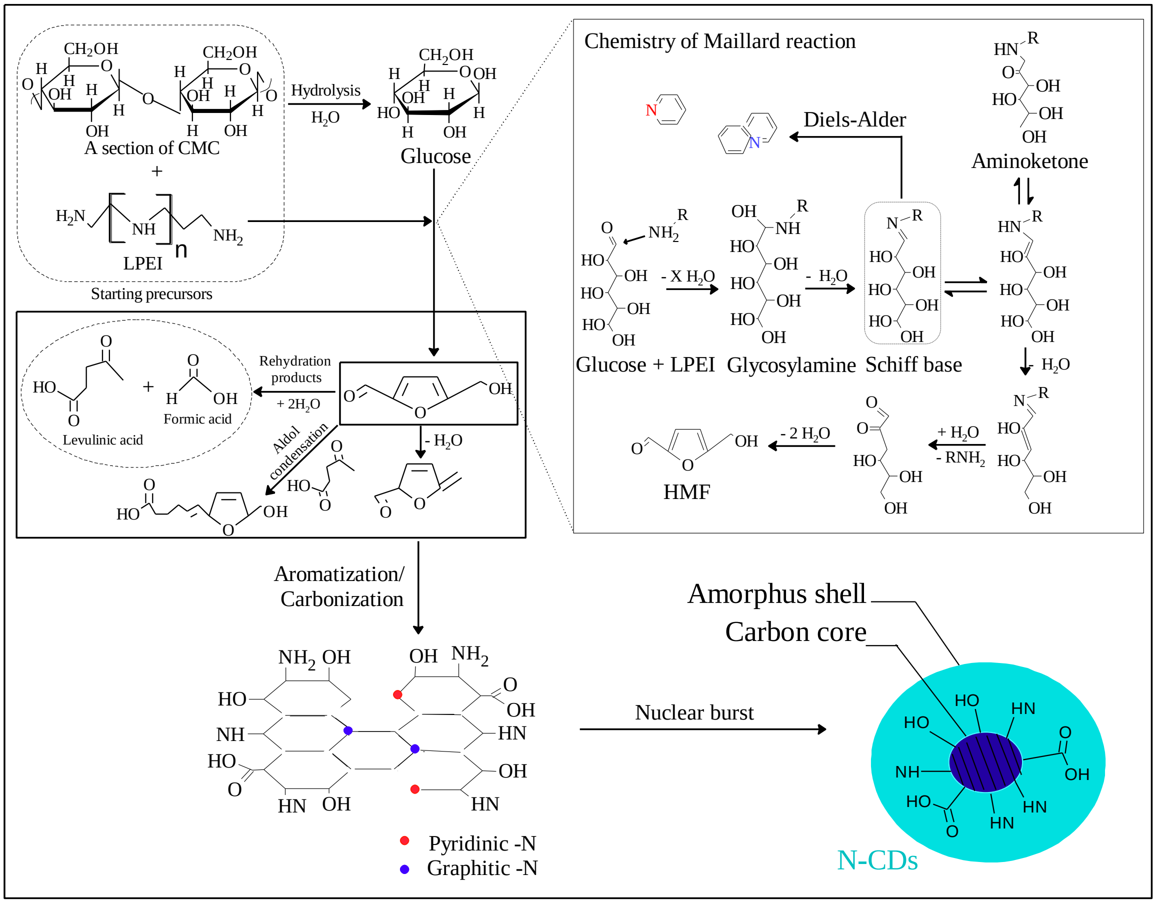


**Fig. S1. Fundamental understanding of the N-CDs formation pathway**

Soluble organic acids such as levulinic and formic acid were formed via rehydration of HMF (the main reaction intermediate in HTC). Various soluble polymers may be formed as a result of fragmentation, substitution, aldol condensation, reversion, and/ or dehydration processes of the acid reaction processes with each other and with some monomers [42]. According to Lamar model, aromatization and carbonization take place by nuclear growth of these aromatic groups. This leads to the formation of polar soluble oxygen/nitrogen containing groups, like -OH, -COOH, $CN^-$ and -NH that can be attached to the surface of CDs, as supported by FTIR and XPS spectra. On the other hand, N atoms, including pyridinic -N and graphitic -N (denoted by red and blue ball, respectively) were also introduced into the polyaromatic structure through long-time HTC process [42]. It is believed that the incorporation of pyridinic and graphitic nitrogen atoms could play the major role for fluorescent enhancement by

introducing defect states in the hexagonal ring system of the N-CD core [43][44]. On the other hand, the existence of oxygen/nitrogen containing groups over the N-CDs surface may induce the energy gaps by creating energy traps, leading to the enhancement of PL structure of N-CDs through radiative recombination of localized electron-hole pairs [43][45].

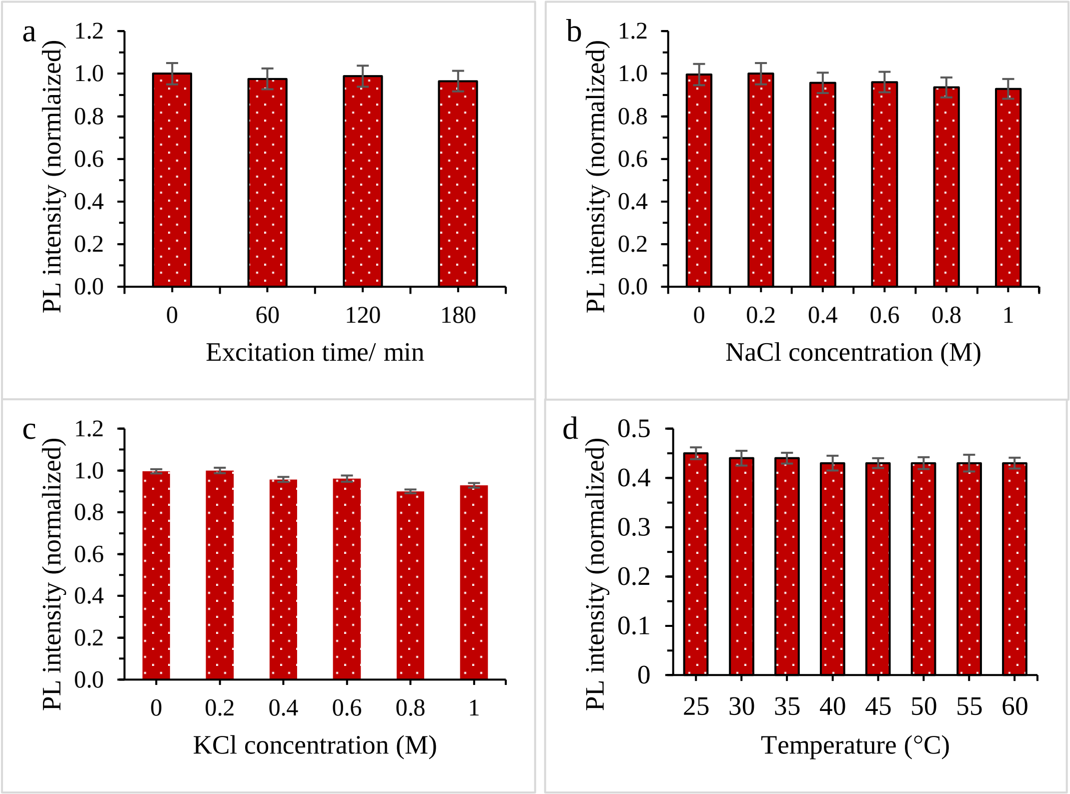


**Fig. S2. PL intensity as a function of (a) illumination time irradiated with UV light (365 nm) and (b) NaCl concentration, (c) KCl concentration and (d) temperatures**

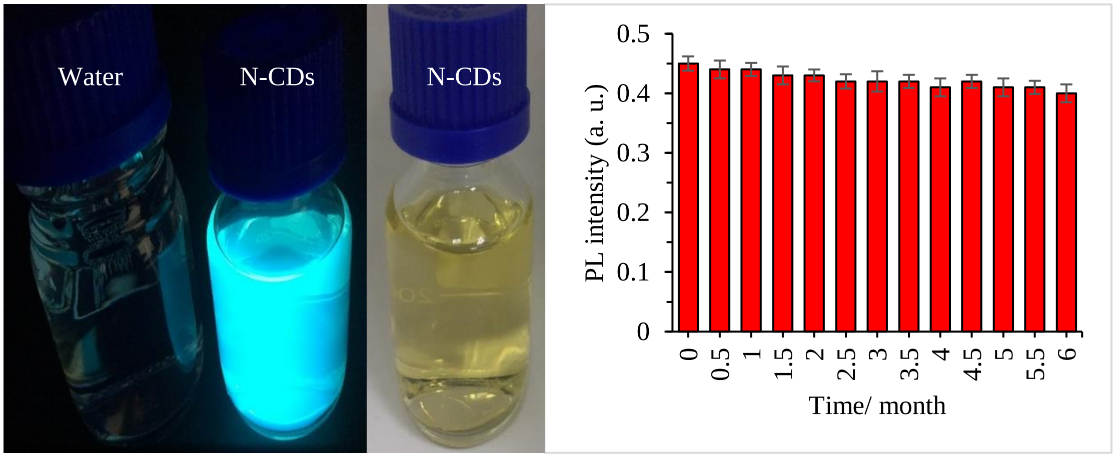

**Fig. S3. (a) Digital images showing N-CDs in ambient light (right) and N-CDs and water (middle and left, respectively) under UV-lamp after six months at room temperature and (b) effect of storage time on the fluorescence intensity of the N-CDs (0-6 months) at 25 °C**

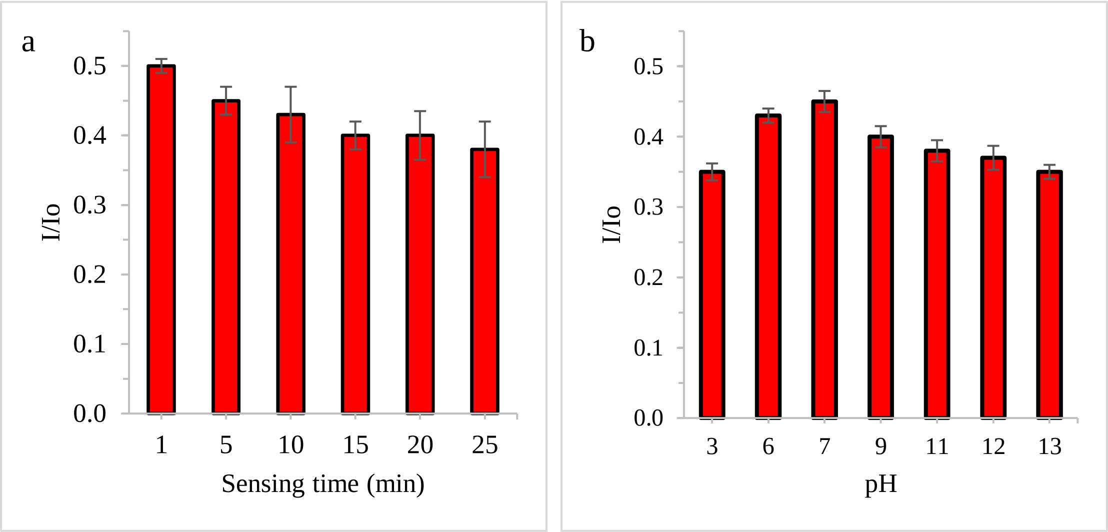


**Fig. S4. The effect of (a) sensing time and (b) pH value on the relative fluorescence quenching of N-CDs before and after addition of 50 μM of copper ions**

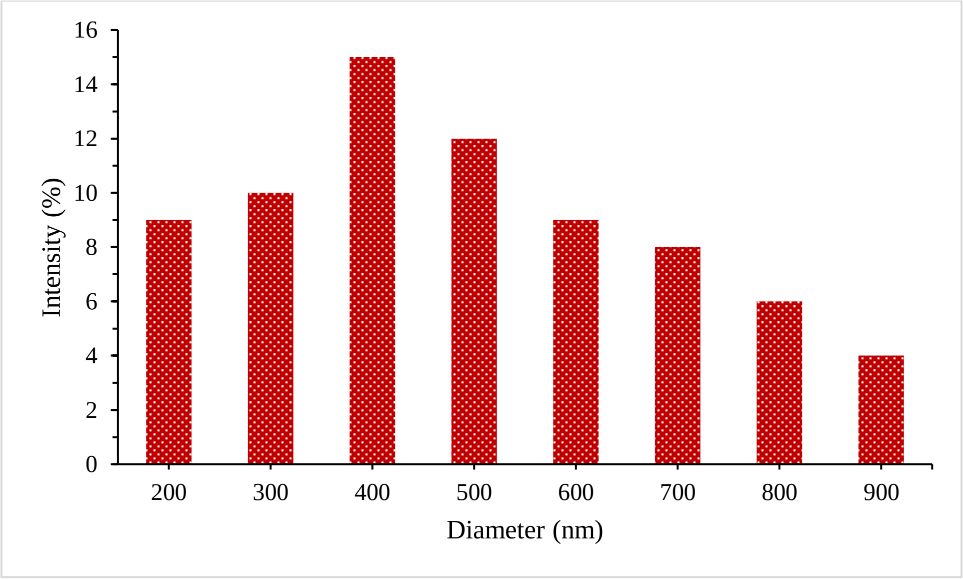


**Fig. S5. DLS size distribution of N-CDs after the addition of 700 μM Cu (II)**

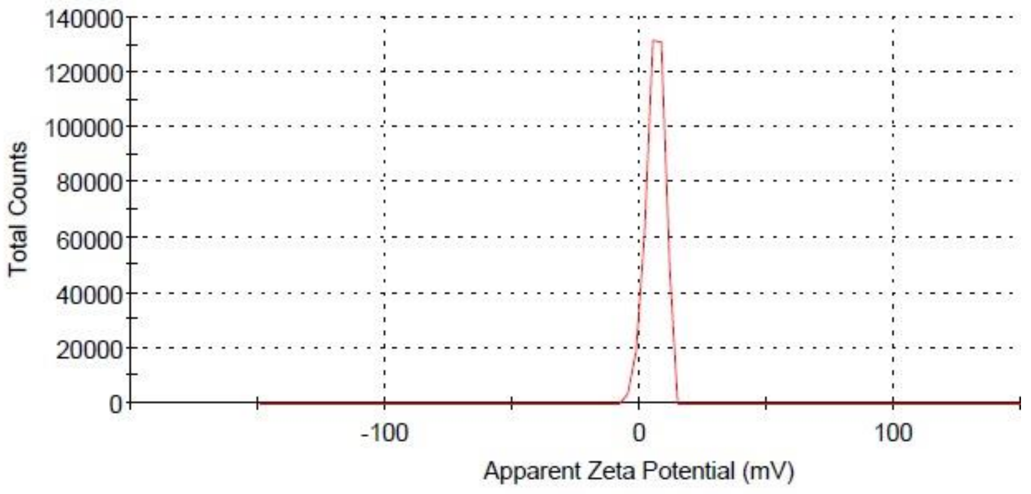


**Fig. S6. Zeta potential of N-CDs after the addition of 700 μM Cu (II)**

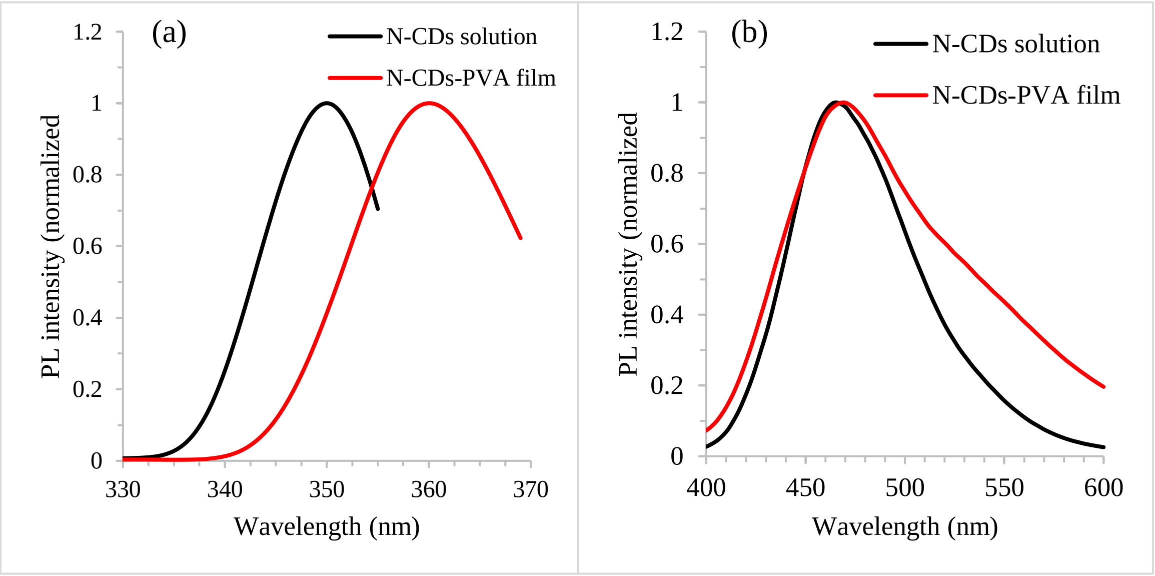


**Fig. S7. PL spectra of N-CDs solution and N-CDs-PVA film showing (a) excitation and (b) emission maxima**

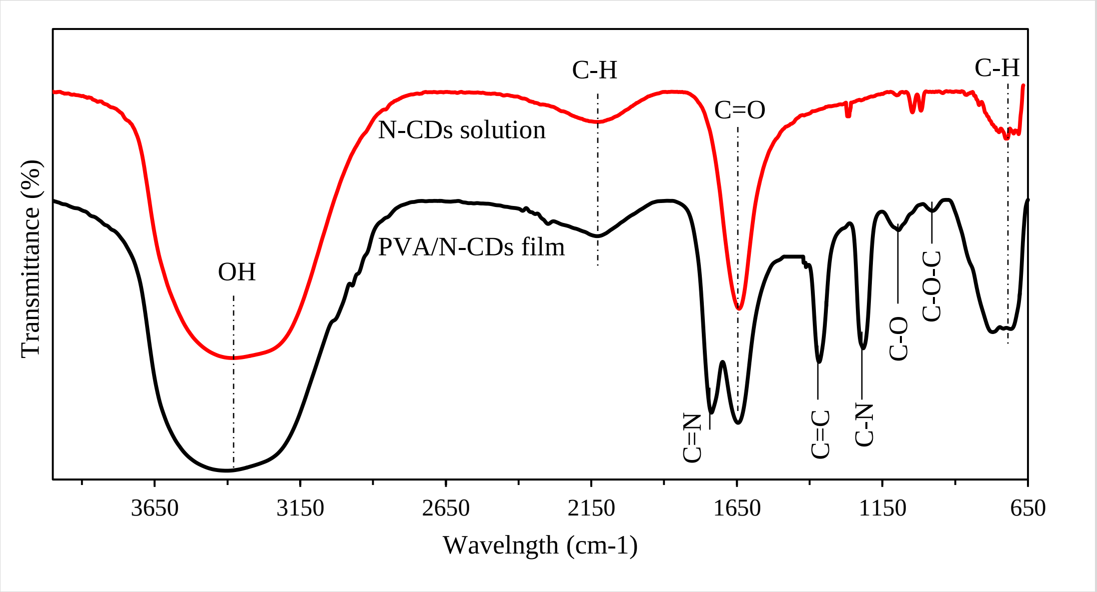


**Fig. S8. FT-IR spectra of the as-synthesized N-CDs (red line) and PVA/N-CDs film (black line)**

**Table S5.** Quantum yield calculation of N-CDs-PVA film

| **Sample** | **Integrated emission intensity (I)** | **Optical density (OD)** | **η** | **Quantum yield (QY)** |
|---|---|---|---|---|
| Quinine sulfate | 35828.9 | 0.033 | 1.33 | 54% (known) |
| N-CDs-PVA film | 37799.1 | 0.04 | 1.33 | 47% |

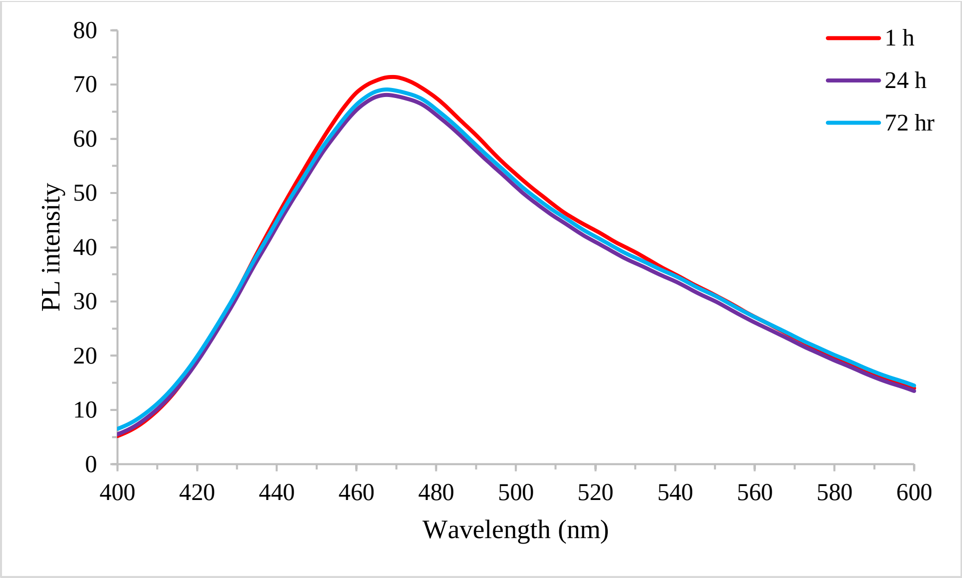


**Fig. S9. Photostability of N-CDs-PVA composite film**

## Reference

[1] B. De and N. Karak, “A green and facile approach for the synthesis of water soluble fluorescent carbon dots from banana juice,” *RSC Adv.*, vol. 3, no. 22, pp. 8286–8290, 2013.

[2] V. N. Mehta, S. Jha, and S. K. Kailasa, “One-pot green synthesis of carbon dots by using Saccharum officinarum juice for fluorescent imaging of bacteria (Escherichia coli) and yeast (Saccharomyces cerevisiae) cells,” *Mater. Sci. Eng. C*, vol. 38, no. 1, pp. 20–27, 2014.

[3] V. N. Mehta, S. Jha, H. Basu, R. K. Singhal, and S. K. Kailasa, “One-step hydrothermal approach to fabricate carbon dots from apple juice for imaging of mycobacterium and fungal cells,” *Sensors Actuators, B Chem.*, vol. 213, pp. 434–443, 2015.

[4] H. Huang, Y. Xu, C.-J. Tang, J.-R. Chen, A.-J. Wang, and J.-J. Feng, “Facile and green synthesis of photoluminescent carbon nanoparticles for cellular imaging,” *New J. Chem.*, vol. 38, no. 2, p. 784, 2014.

[5] R. Purbia and S. Paria, “A simple turn on fluorescent sensor for the selective detection of thiamine using coconut water derived luminescent carbon dots,” *Biosens. Bioelectron.*, vol. 79, pp. 467–475, 2016.

[6] K. H. Wu, J. F. Y. Fong, J. Z. Luk, I. R. Gentle, and D. W. Wang, "Metal-Ligand Complexes as Molecular Metal-Ion Reservoirs for Highly Promoted Growth of β-Co(OH)2Microplates," *Cryst. Growth Des.*, vol. 16, no. 1, pp. 8–11, 2016.

[7] A. Barati, M. Shamsipur, E. Arkan, L. Hosseinzadeh, and H. Abdollahi, "Synthesis of biocompatible and highly photoluminescent nitrogen doped carbon dots from lime: Analytical applications and optimization using response surface methodology," *Mater. Sci. Eng. C*, vol. 47, pp. 325–332, 2015.

[8] A.-M. Alam, B.-Y. Park, Z. K. Ghouri, M. Park, and H.-Y. Kim, "Synthesis of carbon quantum dots from cabbage with down- and up-conversion photoluminescence properties: excellent imaging agent for biomedical applications," *Green Chem.*, vol. 17, no. 7, pp. 3791–3797, 2015.

[9] X. Yang, Y. Zhuo, S. Zhu, Y. Luo, Y. Feng, and Y. Dou, "Novel and green synthesis of high-fluorescent carbon dots originated from honey for sensing and imaging," *Biosens. Bioelectron.*, vol. 60, pp. 292–298, 2014.

[10] S. Han *et al.*, "Fabrication, gradient extraction and surface polarity-dependent photoluminescence of cow milk-derived carbon dots," *RSC Adv.*, vol. 4, no. 101, pp. 58084–58089, 2014.

[11] D. Wang, X. Wang, Y. Guo, W. Liu, and W. Qin, "Luminescent properties of milk carbon dots and their sulphur and nitrogen doped analogues," *RSC Adv.*, vol. 4, no. 93, pp. 51658–51665, 2014.

[12] L. Shi *et al.*, "Naked oats-derived dual-emission carbon nanodots for ratiometric sensing and cellular imaging," *Sensors Actuators, B Chem.*, vol. 210, pp. 533–541, 2015.

[13] X. Qin, W. Lu, A. M. Asiri, A. O. Al-Youbi, and X. Sun, "Microwave-assisted rapid green synthesis of photoluminescent carbon nanodots from flour and their applications for sensitive and selective detection of mercury(II) ions," *Sensors Actuators, B Chem.*, vol. 184, pp. 156–162, 2013.

[14] Q. Liang, W. Ma, Y. Shi, Z. Li, and X. Yang, "Easy synthesis of highly fluorescent carbon quantum dots from gelatin and their luminescent properties and applications," *Carbon N. Y.*, vol. 60, pp. 421–428, 2013.

[15] M. Xue, Z. Zhan, M. Zou, L. Zhang, and S. Zhao, "Green synthesis of stable and biocompatible fluorescent carbon dots from peanut shells for multicolor living cell imaging," *New J. Chem.*, vol. 40, no. 2, pp. 1698–1703,

2016.

[16] W. Lu *et al.*, "Economical, green synthesis of fluorescent carbon nanoparticles and their use as probes for sensitive and selective detection of mercury(II) ions," *Anal. Chem.*, vol. 84, no. 12, pp. 5351–5357, 2012.

[17] M. Tan *et al.*, "Enhanced photoluminescence and characterization of multicolor carbon dots using plant soot as a carbon source," *Talanta*, vol. 115, pp. 950–956, 2013.

[18] Q. Wang, X. Liu, L. Zhang, and Y. Lv, "Microwave-assisted synthesis of carbon nanodots through an eggshell membrane and their fluorescent application.," *Analyst*, vol. 137, no. 22, pp. 5392–7, 2012.

[19] A. Prasannan and T. Imae, "One-pot synthesis of fluorescent carbon dots from orange waste peels," *Ind. Eng. Chem. Res.*, vol. 52, no. 44, pp. 15673–15678, 2013.

[20] J. B. Essner, C. H. Laber, S. Ravula, L. Polo-Parada, and G. A. Baker, "Pee-dots: biocompatible fluorescent carbon dots derived from the upcycling of urine," *Green Chem.*, vol. 18, no. 1, pp. 243–250, 2016.

[21] S. L. D'Souza, B. Deshmukh, J. R. Bhamore, K. A. Rawat, N. Lenka, and S. K. Kailasa, "Synthesis of fluorescent nitrogen-doped carbon dots from dried shrimps for cell imaging and boldine drug delivery system," *RSC Adv.*, vol. 6, no. 15, pp. 12169–12179, 2016.

[22] Q. Dou, X. Fang, S. Jiang, P. L. Chee, T. C. Lee, and X. J. Loh, "Multi-functional fluorescent carbon dots with antibacterial and gene delivery properties," *RSC Adv.*, vol. 5, no. 58, pp. 46817–46822, 2015.

[23] J. Wang *et al.*, "WITHDRAWN: One-pot simple green synthesis of water-soluble cleaner fluorescent carbon dots from cellulose and its sensitive detection of iron ion," *J. Clean. Prod.*, 2017.

[24] H. Li, X. He, Y. Liu, and Z. Kang, "One-step ultrasonic synthesis of water-soluble carbon nanoparticles with excellent photoluminescent properties," *Carbon N. Y.*, vol. 49, no. 2, pp. 605–609, 2010.

[25] P. Shen, J. Gao, J. Cong, Z. Liu, C. Li, and J. Yao, "Synthesis of Cellulose-Based Carbon Dots for Bioimaging," *ChemistrySelect*, vol. 1, no. 7, pp. 1314–1317, 2016.

[26] Q. Wu, W. Li, J. Tan, Y. Wu, and S. Liu, "Hydrothermal carbonization of carboxymethylcellulose: One-pot preparation of conductive carbon

microspheres and water-soluble fluorescent carbon nanodots," *Chem. Eng. J.*, vol. 266, pp. 112–120, 2015.

[27] M. Abdullah Issa *et al.*, "Fabrication, characterization and response surface method optimization for quantum efficiency of fluorescent nitrogen-doped carbon dots obtained from carboxymethylcellulose of oil palms empty fruit bunch," *Chinese J. Chem. Eng.*, 2019.

[28] P. Wu, W. Li, Q. Wu, Y. Liu, and S. Liu, "Hydrothermal synthesis of nitrogen-doped carbon quantum dots from microcrystalline cellulose for the detection of Fe 3+ ions in an acidic environment," *RSC Adv.*, vol. 7, no. 70, pp. 44144–44153, 2017.

[29] L. Wang, Y. Yin, A. Jain, and H. S. Zhou, "Aqueous Phase Synthesis of Highly Luminescent , Nitrogen-Doped Carbon Dots and Their Application as Bioimaging Agents," *Langmuir*, vol. 30, pp. 14270–14275, 2014.

[30] L. Hu *et al.*, "Multifunctional carbon dots with high quantum yield for imaging and gene delivery," *Carbon N. Y.*, vol. 67, pp. 508–513, 2013.

[31] A. Jaiswal, S. S. Ghosh, and A. Chattopadhyay, "One step synthesis of C-dots by microwave mediated caramelization of poly(ethylene glycol)," *Chem. Commun.*, vol. 48, no. 3, pp. 407–409, 2012.

[32] H. Liu, T. Ye, and C. Mao, "Fluorescent carbon nanoparticles derived from candle soot," *Angew. Chemie*, vol. 46, no. 34, pp. 6473–6475, 2007.

[33] Z. A. Qiao *et al.*, "Commercially activated carbon as the source for producing multicolor photoluminescent carbon dots by chemical oxidation," *Chem. Commun.*, vol. 46, no. 46, pp. 8812–8814, 2010.

[34] M. Sevilla and A. B. Fuertes, "The production of carbon materials by hydrothermal carbonization of cellulose," *Carbon N. Y.*, vol. 47, no. 9, pp. 2281–2289, 2009.

[35] K. Yu, J. Wang, K. Song, X. Wang, C. Liang, and Y. Dou, "Hydrothermal Synthesis of Cellulose-Derived Carbon Nanospheres from Corn Straw as Anode Materials for Lithium ion Batteries," *Nanomaterials*, vol. 9, no. 1, p. 93, 2019.

[36] E. García-Bordejé, E. Pires, and J. M. Fraile, "Parametric study of the hydrothermal carbonization of cellulose and effect of acidic conditions," *Carbon N. Y.*, vol. 123, pp. 421–432, 2017.

[37] R. Li, L. Wang, and A. Shahbazi, "A Review of Hydrothermal

Carbonization of Carbohydrates for Carbon Spheres Preparation," *Trends Renew. Energy*, vol. 1, no. 1, pp. 43–56, 2015.

[38] C. Falco, N. Baccile, and M. M. Titirici, "Morphological and structural differences between glucose, cellulose and lignocellulosic biomass derived hydrothermal carbons," *Green Chem.*, vol. 13, no. 11, pp. 3273–3281, 2011.

[39] N. R. Pires, C. M. W. Santos, R. R. Sousa, R. C. M. De Paula, P. L. R. Cunha, and J. P. A. Feitosa, "Novel and fast microwave-assisted synthesis of carbon quantum dots from raw cashew gum," *J. Braz. Chem. Soc.*, vol. 26, no. 6, pp. 1274–1282, 2015.

[40] W. J. Niu, Y. Li, R. H. Zhu, D. Shan, Y. R. Fan, and X. J. Zhang, "Ethylenediamine-assisted hydrothermal synthesis of nitrogen-doped carbon quantum dots as fluorescent probes for sensitive biosensing and bioimaging," *Sensors Actuators, B Chem.*, vol. 218, pp. 229–236, 2015.

[41] K. pathy, "Process for Preparation of Vitamin C and Method for Determination of Vitamin C in Tablets," *Surg. Case Stud. Open Access J.*, vol. 1, no. 3, pp. 1–14, 2018.

[42] J. Yang, W. Chen, X. Liu, Y. Zhang, and Y. Bai, "Hydrothermal synthesis and photoluminescent mechanistic investigation of highly fluorescent nitrogen doped carbon dots from amino acids," *Mater. Res. Bull.*, vol. 89, pp. 26–32, 2017.

[43] V. Arul and M. G. Sethuraman, "Facile green synthesis of fluorescent N-doped carbon dots from Actinidia deliciosa and their catalytic activity and cytotoxicity applications," *Opt. Mater. (Amst).*, vol. 78, pp. 181–190, 2018.

[44] C. J. Reckmeier, J. Schneider, A. S. Susha, and A. L. Rogach, "Luminescent colloidal carbon dots: optical properties and effects of doping [Invited]," *Opt. Express*, vol. 24, no. 2, p. A312, 2016.

[45] L. Li and T. Dong, "Photoluminescence Tuning in Carbon Dots: Surface Passivation or/and Functionalization, Heteroatom Doping," *J. Mater. Chem. C*, vol. 6, no. 30, pp. 7944–7970, 2018.
